# Modern Electrical Methods as a Substitute for Surgery in Certain Pelvic Affections

**Published:** 1892-11

**Authors:** G. Betton Massey

**Affiliations:** Philadelphia, Pa.


### MODERN ELECTRICAL METHODS AS A SUBSTITUTE FOR SURGERY IN CERTAIN PELVIC AFFECTIONS. *

BY G. BETTON MASSEY, M. D.,
PHILADELPHIA, PA.

The first subject considered was the treatment of conditions for which wholesale castration is now practiced, the class of cases alluded to being defined as that generally spoken of by the patients themselves as having diseased ovaries ; at least that is the diagnosis mentioned by the patient as having been made by Doctor So-and-so, who either told her that an operation was necessary, or had postponed it for a few weeks, during which a half-hearted attempt to avert it is made. The diagnosis made by the surgeon was doubtless a salpingo-oöphoritis, the frequent existence of which the reader admitted, but wished to be understood as asserting that the case is rare in which a salpingo-oöphoritis is the true diagnosis in the sense that it is the most important feature of the disease with which the patient is suffer-

*Abstract of a paper read (by invitation) before the Virginia State Medical Society, September, 1892.

ing. What is the condition of the uterus in these cases? His investigations had nearly always pointed to the continued existence of the primary inflammation in the uterus in conjunction with the later developed tubo-ovarian mischief, the uterine inflammation remaining the most important element in the case. In other words, the conventional diseased ovary is a myth rather than an affection of primal importance, the graver cases of cystoma, abscess and tuberculosis excepted. He did not deny that inflamed ovaries were daily removed by abdominal section, but the important fact remained that the patients from whom they are removed, when they recover from the operation, are rarely in better health afterwards, and are often made worse. To an inflamed and hypertrophied uterus, to which they owed mainly their original suffering, was added the suffering from cut and cicatrized nerves, painful stumps, bowel adhesions and irregular climacterics, not to mention the peculiar effects on the nervous system so often following castration.

The reader of the paper made a plea for the recognition and proper treatment of the metritis in these cases, and claimed that the best mode of treating this element in the case is by electricity, for the reason that we are not possessed of any other agent that will, at one and the same time, combat the microbic catarrh, promote absorption, contract the uterine muscular tissue, and improve the nutrition of the part. A case of a young lady was cited in which two eminent operators advised section, but who was sent to Dr. Massey by her family physician. The ovaries were found to be of normal size, though movable, and by no means as tender as the uterus, which was enlarged and catarrhal. Ten weeks' treatment removed nearly all of the suffering and restored the patient to health, and the progress of the case demonstrated clearly the value of the intra-uterine applications.

The reader's experience in the electrical treatment of fibroids was related, embracing sixty-eight cases. Of this number seven disappeared entirely by electrically induced absorption. Including these, sixty-four were practically cured of pain, hemorrhage, and pressure symptoms, nearly all of which were reduced in size to varying degrees. The best results are not to be attained by triflers, jacks-of-all-trades, or surgeons who wish to nurse a case for operation; nor even by the most conscientious man who is unfitted for the attainment of expertness in the method by reason of an ambition to shine as an abdominal surgeon, for it is a work demanding as much devotion as any other in the present limits of specialism. In properly selected cases, 100 per cent. of practical cures are attainable. It should commend itself to physician and patient alike, on account of the certainty of attaining at least a permanent arrest of the growth, relief from suffering, and some reduction in size, together with a possibility of a great reduction and even total disappearance. Abnormally soft and cystic tumors were not amenable to the treatment. Though size was no bar to success, the profession should begin to find the small tumors and place them under treatment, instead of mistaking them for displacements, etc.

The lately recognized value of electricity in the treatment of displacements was due to the fact that chronic metritis from catarrhal troubles was the real trouble, as in the so-called ovarian cases. Increased knowledge of the microbic origin of hyperplasias, and the secondary deviations and versions of the abnormally heavy organ, would correct the prevalent habit of using pessaries for these affections, the proper treatment being directed to the primal enlargement and inflammation, which should be combatted, the normal muscular tone and local tissue activities being also restored by the use of both currents.

